# Healthcare, Insurance, and Medical Expenditure of the Floating Population in Beijing, China

**DOI:** 10.3389/fpubh.2020.00375

**Published:** 2020-08-06

**Authors:** Chenjin Ma, Yuming Zhang, Yang Li, Yu Wang, Yan Jiang, Xiaojun Wang, Shuangge Ma

**Affiliations:** ^1^School of Statistics, Renmin University of China, Beijing, China; ^2^Center for Applied Statistics, Renmin University of China, Beijing, China; ^3^Statistical Consulting Center, Renmin University of China, Beijing, China; ^4^School of Public Health, Yale University, New Haven, CT, United States

**Keywords:** floating population, China, healthcare, insurance, medical expenditure

## Abstract

**Background:** China has a large floating population created by the fast urbanization and unique hukou system. With low socioeconomic status, labor-intensive jobs, and the lack of portability of health insurance, the floating population are often disadvantageous in healthcare. However, there is often insufficient attention to healthcare of the floating population.

**Method:** To provide an informative description of certain aspects of the floating population under healthcare, particularly including demographic characteristics, illness conditions, insurance utilization, and medical expenditure, a survey study was conducted in Beijing, China, collecting data on 437 subjects. Characteristics of the floating population and treatments of their illness conditions are examined using univariate and multivariate regression analysis.

**Results:** Personal characteristics and healthcare of the floating population are examined in detail. It is found that the floating population has low insurance coverage and utilization rates. Multiple personal characteristics are identified as significantly associated with insurance utilization and medical expenditure.

**Conclusions:** This study suggests the necessity of further improving healthcare and health insurance protection for the floating population. The identified significant characteristics may assist healthcare providers and other stakeholders identifying the less advantaged.

## Background

With fast urbanization, China has been facing a unique floating population problem in the past two decades. Although multiple definitions exist in the literature (1–3), the most commonly accepted is the one by the Census 2000, which defines the floating population as “individuals who have resided at the place of destination for at least 6 months without local household registration status” (4). As can be partly seen from this definition, the uniqueness of the Chinese floating population is strongly associated with the “hukou” (household registration) system in China. According to the “China's floating population development report 2016” issued by the National Health and Family Planning Commission Mobile Population Service Center (5), by the end of 2014, the size of the floating population was about 253 million, roughly 18% of the total population in China, which is considerably larger than most other social groups. A consistent growth of the floating population was observed between 2011 and 2014. It is expected that in the near future, the size of the floating population will remain large.

The floating population in China shares some similarity with their counterparts—often referred to as “migrant workers”—in other countries including the U.S. (6–9). The dominating majority of China's floating population come from rural areas with low economic status, such as the Sichuan, Anhui, and Henan Provinces. Most of them are young and not well-educated. They usually work in labor-intensive industries, such as manufacturing, hotel, and catering, services, and others. It has been well-recognized in the literature that, with often poor working conditions, low socioeconomic status, and other factors, migrant workers are disadvantageous in healthcare (10–12). China's floating population also faces unique challenges, which are largely associated with the unique health insurance system. The basic insurance system offered by the government consists of three schemes: UEBMI (Urban Employee Basic Medical Insurance, for the employed in the urban areas), URBMI (Urban Resident Basic Medical Insurance, for urban residents not covered by the UEBMI), and NCMS (New Rural Cooperative Medical Scheme, for the rural residents). Extensive discussions on this three-component insurance system are available in the literature (13, 14). Those who migrate from rural to urban areas, which are the majority of the floating population, are entitled to the NCMS at their hometown. However, the NCMS has poor portability. In particular, to be eligible for insurance reimbursement for healthcare at the live/work place, one has to get pre-approval and also apply for reimbursement at his/her hometown (as opposed to where treatment happens). Such a cumbersome procedure often results in poor protection for the floating population at their live/work places.

In the literature, multiple studies on the healthcare of migrant workers have been conducted (15–17). For example, Moyce and Schenker (18) showed that the incidence of adverse occupational exposure and working conditions among migrant workers is higher worldwide, leading to poor health outcomes, workplace injuries, and occupational fatalities. Studies have characterized the role of migration and social movement in the spread of HIV and STIs both nationally and internationally (19–21). Hu et al. (22) summarized the three main concerns on migrant health: infectious diseases, maternal health and occupational diseases, and injuries. A cross-sectional study in the Jiangsu Province, China identified multiple predictors for whether the floating population received social insurance (23). A semi-structured in-depth interview conducted in Tianjin, China reviewed that, despite significant effort in policy and social interventions, the floating population were still, in many respects, not integrated into the urban society (17). Recent studies on the Asian migrant populations showed that migrant workers with high acculturative stress were more likely to have mental health problems and less likely to engage in health-seeking behaviors (24–26).

Our literature review suggests that, compared to the general population and some other social groups, research on the healthcare of the floating population in China is significantly more limited. Considering its uniquely large size, research on the healthcare of the floating population can have high public health value. Most of the existing studies have been focused on the policy aspects (for example, the design of an insurance system with better portability) (27), management (28), specific diseases (especially work-related) (29), and specific types of disease treatment (30). The goal of this study is to directly collect and analyze empirical data from the floating population, and to provide an updated and detailed description of multiple aspects of the healthcare of China's floating population. Specifically, we first examine demographic characteristics under different treatments to gain more insights into the basic characteristics of the floating population with illness conditions. We then examine insurance utilization, which has been motivated by the poor portability of health insurance observed in the literature. Published studies have also suggested that the floating population is significantly and negatively affected by the collective effect of high medical cost, poor insurance portability, disadvantageous working conditions, and low income. To gain more insights into this aspect, we pursue the analysis of medical cost. It is expected that this study may provide valuable insights into this unique population, which may facilitate healthcare providers and other stakeholders to further improve healthcare of the floating population. With a different perspective, this study may complement the existing studies especially those on policy and macro management.

## Methods

### Data Collection

This study was conducted as a part of the CSPH (China Survey on Pension and Healthcare), which is a collaborative effort by the Renmin University of China (RUC) and Yale School of Public Health. It was approved by an ethics review committee at the RUC. A survey was conducted in Oct, 2014 in Beijing, which has one of the largest floating population in China and is a representative of the highly-developed and populated urban areas. Studies have suggested that characteristics of the floating population in Beijing are very similar to those in major cities such as Shanghai, Shenzhen, and Guangzhou (31). Beijing has a total of ten districts, among which six were selected with three (Chaoyang, Haidian, and Xicheng) having above-median per capita GDP and three (Fengtai, Changping, and Tongzhou) below-median. Within each district, a stratified sampling approach was adopted to achieve representativeness.

At the beginning of each survey, the interviewer introduced the nature and purpose of the survey and collected basic information. An interviewee was qualified if he/she was at least 18 years old, had resided in Beijing for at least 6 months but with “hukou” in a different city/province, and had at least one disease episode in a period of 12 months prior to survey. Each interviewee who agreed to participate signed an informed consent form. Basic information on the non-responders was collected and analyzed, and no significant differences were found between the responders and non-responders.

The survey consists of two sections. The first is on subject's characteristics, including gender, age, marital status, education, occupation, type of household (hukou), physical condition, health insurance status, individual, and household income, and expenditure. Such information has been routinely collected in peer studies. The second section is on healthcare, including inpatient, outpatient, and self-treatments. Detailed information is collected on disease under treatment, health insurance utilization, and medical expenditure. It is noted that a treatment episode may broadly include both allopathic/orthodox treatments as well as alternative medicine such as traditional Chinese medicine, which is popular in China. However, with constrained resources (which limit how many questions can be asked in the survey), information on specific diagnosis and treatment strategies is not collected. Although the significance of such information, for example for insurance utilization, cost, and end results, is fully acknowledged, it is comparatively less important than other information, for example the presence of treatment. We also note that in some peer studies, information has also been collected on cultural, and religious information which may affect healthcare behaviors. For the surveyed population, cultural differences are small, and the dominating majority do not have religious beliefs. It is also noted that many peer studies also do not include cultural and religious information (15, 16, 32). More details on the collected information are provided below and in the tables.

### Data Analysis

In the first set of analysis, subjects' characteristics and disease conditions for the whole cohort as well as subgroups with each type of treatment were examined. This analysis can characterize the study cohort. In the second set of analysis, insurance coverage and utilization were examined. For a specific type of treatment, analysis was conducted to identify personal characteristics associated with insurance utilization. As described above, there exist major differences in insurance between China's floating population and their counterparts in other countries. This set of analysis can quantify the insurance utilization characteristics of China's floating population. In the third set of analysis, medical expenditure was examined. Here two types of cost were analyzed. The first is total cost, defined as the sum of treatment cost and lost income. The second is OOP (out of pocket) cost, defined as the total cost minus insurance payment (if insurance utilized). This set of analysis can identify personal characteristics associated with high medical cost. Associating personal characteristics and health conditions with insurance utilization and medical cost has been conducted in quite a few published studies and suggested as having important implications. It is also noted that insurance and healthcare pursuit behaviors are very complicated, and there is still a lack of consensus on what variables may be more/less relevant, especially for this specific population. As such, there may be relevant variables missed in our survey.

In all three sets of analysis, summary statistics were computed. Specifically, for categorical variables, counts, and percentages were computed, and comparisons across groups were made using Chi-squared and Fisher tests. For continuous variables approximately normally distributed, means, and standard deviations were computed, and comparisons were made using *t*-tests. For continuous variables with skewed distributions, medians, and MADs (median absolute deviations) were computed, and comparisons were made using Wilcoxon tests. In the second and third sets of analysis, multivariate regressions were conducted. For insurance utilization which has a binary response, logistic regression was applied. For medical cost which has a continuous response, linear regression was conducted. To accommodate skewed (non-normal) distributions, the LAD (least absolute deviation) estimation was adopted, and so transformation, which is adopted in some published cost studies, was not pursued. To accommodate small sample sizes and improve estimation stability, we adopted a step-wise approach, and the final models contained only effects that are significant. For insurance utilization with inpatient treatment which has an extremely small sample size, *p*-value cutoff 0.1 was used. In other regression analyses, *p*-value cutoff 0.05 was used. Extensive model examinations on collinearity, heteroskedasticity, model specification, and several other aspects were conducted using graphical and hypothesis testing techniques, and no serious violation was identified. All analyses were conducted using R 3.4.4.

## Results

### Subjects' Characteristics

A total of 437 subjects finished the survey, with a response rate of 62% which is comparable to peer studies. Detailed results are shown in Table 1. Among the surveyed subjects, 57.7% are female. Most are young (51.8% in the 18–30 age group), married (64.3%), and not well-educated (only 7.8% with college or above education). The three dominating occupations are hotel and catering (33.9%), service (29.8%), and sales (30.4%). Most have their hukou as rural (69.3%) and are relatively healthy (90.6% healthy or just so-so). The dominating majority have insurance (86.3%), however, most are at their hometown (77.6%) not Beijing (21.1%). On average, they had stayed in Beijing for 11.1 years. The average annual personal income is 40.0 K RMB.

**Table 1:** Characteristics of the whole cohort and subgroups with different types of treatment.

	**All subjects**	**Treatment**		
	**(*n =* 437)**			
		**Inpatient**	**Outpatient**	**Self-treatment**
		**(*n =* 54)**	**(*n =* 269)**	**(*n =* 379)**
**Gender**				
Male	185 (42.3)	30 (55.6)	106 (39.2)	157 (41.4)
Female	252 (57.7)	24 (44.4)	163 (60.8)	222 (58.6)
**Age group**				
18–30	226 (51.8)	22 (40.7)	148 (55.2)	188 (49.7)
30–40	81 (18.6)	14 (25.9)	45 (16.8)	75 (19.8)
40–50	89 (20.4)	11 (20.4)	54 (20.1)	80 (21.2)
50–60	33 (7.6)	5 (9.3)	17 (6.3)	28 (7.5)
>60	7 (1.6)	2 (3.7)	4 (1.4)	7 (1.9)
**Marital status**				
Single, divorced, widowed	156 (35.7)	15 (27.8)	105 (39.0)	135 (35.6)
Married	281 (64.3)	39 (72.2)	164 (61.0)	244 (64.4)
**Education**				
No schooling	17 (4.0)	2 (3.8)	9 (3.5)	15 (4.1)
Primary	41 (9.7)	7 (13.2)	22 (8.5)	38 (10.4)
Junior high	154 (36.4)	9 (17.0)	86 (33.2)	134 (36.5)
Senior high	115 (27.2)	14 (26.4)	79 (30.5)	99 (27.0)
Junior college	63 (14.9)	9 (17.0)	40 (15.4)	54 (14.7)
College and more	33 (7.8)	12 (22.6)	23 (8.9)	27 (7.3)
**Occupation**				
Manufacturing	7 (1.6)	3 (5.6)	6 (2.2)	5 (13.2)
Hotel and catering	148 (33.9)	10 (18.5)	81 (30.1)	135 (35.6)
Service	130 (29.8)	16 (29.6)	80 (29.8)	118 (31.1)
Wholesale and retail	133 (30.4)	15 (27.8)	95 (35.3)	111 (29.3)
Construction	8 (1.8)	3 (5.6)	3 (1.1)	6 (1.6)
Unemployed	4 (0.9)	1 (1.9)	2 (0.7)	2 (0.5)
Other	7 (1.6)	6 (11.1)	2 (0.7)	2 (0.5)
**Type of household**				
Urban	134 (30.7)	19 (35.2)	84 (31.2)	117 (30.9)
Rural	303 (69.3)	35 (64.8)	185 (68.8)	262 (69.1)
**Physical condition**				
Healthy	272 (62.2)	19 (35.2)	159 (59.1)	237 (62.5)
Just so-so	124 (28.4)	16 (29.6)	82 (30.5)	111 (29.3)
Slightly sick	25 (5.7)	10 (18.5)	20 (7.4)	19 (5.0)
Sick	12 (2.8)	7 (13.0)	4 (1.5)	9 (2.4)
Seriously sick	4 (0.9)	2 (3.7)	4 (1.5)	3 (0.8)
**Health insurance**				
Yes	377 (86.3)	46 (85.2)	229 (85.1)	322 (85.0)
No	60 (13.7)	8 (14.8)	40 (14.9)	57 (15.0)
**Hometown health insurance**				
Yes	339 (77.6)	40 (74.1)	204 (75.8)	291 (76.8)
No	98 (22.4)	14 (25.9)	65 (24.2)	88 (23.2)
**Beijing health insurance**				
Yes	92 (21.1)	11 (20.4)	63 (23.4)	76 (20.1)
No	345 (78.9)	43 (79.6)	206 (76.6)	303 (79.9)
**Time in Beijing (years)**	11.1 ± 1.8	11.2 ± 1.9	11.1 ± 1.7	11.2 ± 1.7
**Individual income (K RMB)**	40.00 (29.65)	40.00 (26.69)	40.00 (29,65)	40.00 (23.72)
**Family income (K RMB)**	86.00 (53.37)	78.50 (44.48)	100.00 (74.13)	80.00 (53.37)
**Medical expenditure (K RMB)**	1.50 (1.78)	14.75 (17.42)	2.00 (2.08)	1.25 (1.26)
**Total spending (K RMB)**	35.00 (22.24)	53.50 (33.36)	35.00 (22.24)	32.00 (23.72)
**Health insurance utilization**				
Yes	—	17 (31.5)	16 (5.9)	7 (1.9)
No	—	37 (68.5)	253 (94.1)	372 (98.1)

*For a categorical variable, count (percentage); For a continuous variable approximately normally distributed, mean ± standard deviation; For a continuous variable with a skewed distribution, median (MAD)*.

Among the surveyed subjects, 54 (12.4%), 269 (61.6%), and 379 (86.7%) had inpatient, outpatient, and self-treatments in a period of 12 months prior to the survey. Differences are observed across the three treatment groups, as well as between those with and without treatments. For example, those with outpatient treatments have more females, are younger, and have a lower percentage of being married. Those with inpatient treatments have a higher a percentage of urban hukou and the lowest percentage of physical condition being healthy. They also have the lowest family income but the highest medical expenditure and total expenditure.

### Diseases Under Treatments

**Figure 1:**
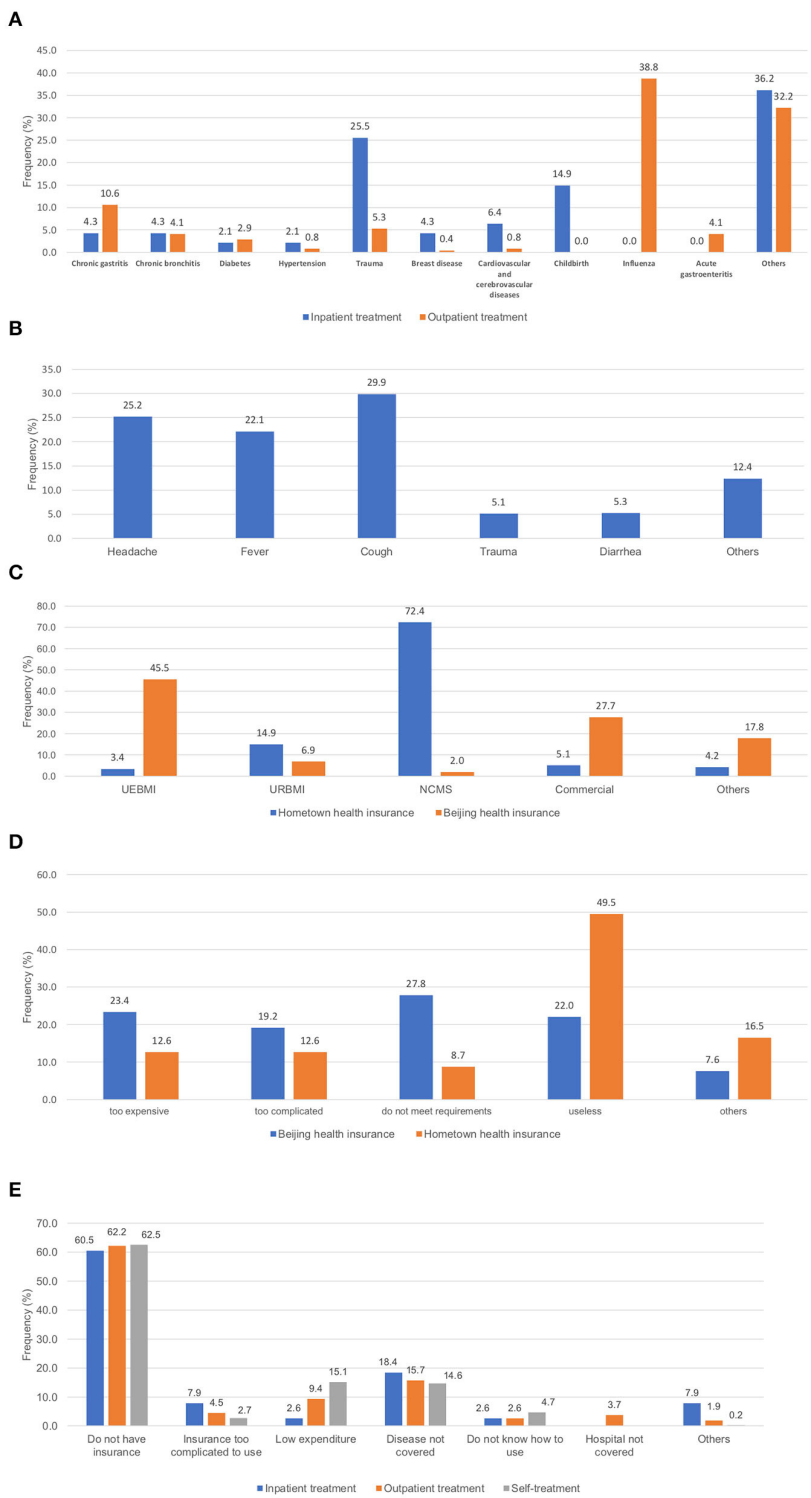
**(A)** Diseases under inpatient and outpatient treatments; **(B)** disease under self-treatments; **(C)** insurance types at hometown and Beijing; **(D)** reasons for not having insurance; **(E)** reasons for not using insurance.

The diseases under different types of treatments are presented in Figures 1A,B. For inpatient treatment, except for the “others” category, the leading conditions are trauma (25.5%) and childbirth (14.9%). For outpatient treatment, the leading conditions are influenza (38.8%) and chronic gastritis (10.6%). And for self-treatment, the leading conditions are cough (29.9%), headache (25.2%), and fever (22.1%).

### Insurance Coverage and Utilization

Results on insurance coverage are presented in Figure 1C. At their hometown, the dominating insurance type for the floating population is NCMS (72.4%), followed by URBMI (14.9%), while other categories have very small percentages. At Beijing, the largest category is UEBMI (45.5%), followed by commercial insurance (27.7%). The reasons for not having insurance are analyzed, and the results are presented in Figure 1D. For insurance at hometown, the most prominent reason is that “insurance is useless” (49.5%), followed by “too expensive” (12.6%) and “too complicated” (12.6%). For insurance at Beijing, four reasons, namely “do not meet requirements” (27.8%), “too expensive” (23.4%), “insurance is useless” (22.0%), and “too complicated” (19.2%), are important contributing factors.

As observed in the literature, high insurance coverage does not imply high utilization. Table 1 shows that the rate of insurance utilization is very low. Specifically, for the three types of treatment, the rates are 31.5, 5.9, and 1.9%, respectively. The reasons for not using insurance are examined, and the results are presented in Figure 1E. For all three types of treatment, “do not have insurance” and “disease not covered” are the most prominent reasons. There are also treatment type-specific reasons, for example “low expenditure” for self-treatment (15.1%) and “insurance too complicated” for inpatient treatment (7.9%).

For each type of treatment separately, univariate analysis is conducted, comparing the group that used insurance with that did not. Results are presented in Table 2. Multiple significant differences are observed. For inpatient treatment, education is observed to distribute significantly differently. Specifically, those who used insurance have significantly higher education levels, for example, 52.9% with college and more, compared to 8.5% for those who did not use insurance. For outpatient treatment, education is also significant (*p*-value 0.027), and the pattern is similar to that for inpatient treatment. In addition, occupation is also observed to be significant. Among those who used insurance, “service” has a much higher percentage (50%) than other categories, whereas the distribution of occupation is “more even” among those who did not use insurance. Those who used insurance are also found to have higher family income (150.0 vs. 96.0 K, *p*-value 0.016), higher treatment cost (2.5 vs. 0.7 K, *p*-value 0.003), and higher gross total cost (3.0 vs. 1.0 K, *p*-value 0.014). In the analysis of self-treatment, no variable is found to be significant.

**Table 2:** Summary statistics for treatment episodes with different insurance utilization status.

	**Insurance utilization**								
	**Inpatient treatment**			**Outpatient treatment**			**Self-treatment**		
	**Yes**	**No**	***p***	**Yes**	**No**	***p***	**Yes**	**No**	***p***
	***n =* 17**	***n =* 37**		***n =* 16**	***n =* 253**		***n =* 7**	***n =* 372**	
**Gender**									
Male	10 (58.8)	20 (54.1)	0.777	4 (25.0)	102 (40.5)	0.295	2 (28.6)	156 (41.8)	0.827
Female	7 (41.2)	17 (45.9)		12 (75.0)	151 (59.7)		5 (71.4)	216 (58.2)	
**Age group**									
18–30	6 (35.3)	16 (43.2)	0.884	10 (62.5)	138 (54.5)	0.605	4 (57.1)	184 (49.5)	0.943
30–40	5 (29.4)	9 (24.3)		1 (6.3)	44 (17.4)		2 (28.6)	73 (19.6)	
40–50	4 (20.4)	7 (18.9)		3 (18.8)	51 (5.9)		1 (14.3)	79 (21.3)	
50–60	2 (3.7)	3 (8.1)		2 (12.5)	4 (1.6)		0 (0.0)	28 (7.6)	
>60	0 (0.0)	2 (5.4)		0 (0.0)	1 (0.4)		0 (0.0)	7 (2.0)	
**Marital status**									
Single, divorced, widowed	4 (23.5)	11 (29.7)	0.751	7 (43.8)	98 (38.7)	0.793	2 (28.6)	133 (35.8)	0.999
Married	13 (76.5)	26 (70.3)		9 (56.3)	155 (61.3)		5 (71.4)	239 (64.2)	
**Education**									
No schooling	1 (5.9)	1 (3.1)	0.003	0 (0.0)	9 (3.7)	0.027	0 (0.0)	15 (4.5)	0.322
Primary school	2 (13.0)	5 (13.9)		1 (6.3)	21 (8.4)		0 (0.0)	38 (15.3)	
Junior high	1 (5.9)	8 (22.1)		3 (18.8)	83 (32.9)		2 (28.6)	132 (40.6)	
Senior high	4 (23.5)	10 (27.5)		3 (18.8)	76 (30.1)		1 (14.3)	98 (30.3)	
Junior college	0 (0.0)	9 (24.9)		3 (18.8)	37 (14.6)		2 (28.6)	52 (19.0)	
College and more	9 (52.9)	3 (8.5)		6 (37.5)	17 (6.7)		2 (28.6)	25 (11.7)	
**Occupation**									
Manufacturing	1 (5.9)	2 (5.4)	0.754	1 (6.3)	5 (2.0)	0.048	0 (0.0)	5 (1.3)	0.841
Hotel and catering	4 (23.5)	6 (16.2)		4 (25.0)	77 (30.4)		3 (42.9)	132 (35.5)	
Service	5 (29.4)	11 (29.7)		8 (50.0)	72 (28.5)		2 (28.6)	116 (31.2)	
Sales	3 (17.6)	12 (32.4)		2 (12.5)	93 (36.8)		2 (28.6)	109 (29.3)	
Construction	2 (11.8)	1 (2.7)		0 (0.0)	3 (1.2)		0 (0.0)	6 (1.6)	
Unemployed	0 (0.0)	1 (2.7)		0 (0.0)	2 (0.8)		0 (0.0)	2 (0.5)	
Other	2 (11.8)	4 (10.8)		1 (6.3)	1 (0.4)		0 (0.0)	2 (0.5)	
**Type of household**									
Urban	9 (52.9)	10 (27.0)	0.076	8 (50.0)	76 (30.0)	0.103	4 (57.1)	113 (30.4)	0.136
Rural	8 (47.1)	27 (73.0)		8 (50.0)	177 (70.0)		3 (42.9)	259 (69.6)	
**Physical condition**									
Healthy	7 (41.2)	12 (32.4)	0.705	13 (81.3)	146 (57.7)	0.454	5 (71.4)	232 (62.4)	0.954
Just so-so	4 (23.5)	12 (32.4)		2 (12.6)	80 (31.6)		2 (28.6)	109 (29.3)	
Slightly sick	2 (11.8)	8 (21.6)		1 (6.3)	19 (7.5)		0 (0.0)	19 (5.1)	
Sick	3 (17.6)	4 (10.8)		0 (0.0)	4 (1.6)		0 (0.0)	9 (2.4)	
Seriously sick	1 (5.9)	1 (2.7)		0 (0.0)	4 (1.6)		0 (0.0)	3 (0.8)	
**Type of hospital**									
Grade I	0 (0.0)	0 (0.0)	0.535	1 (7.1)	34 (13.5)	0.330	—	—	—
Grade II	6 (37.5)	12 (32.4)		6 (42.9)	89 (35.3)				
Grade III	10 (62.5)	24 (64.9)		7 (50.00)	90 (35.7)				
Private	0 (0.0)	1 (2.7)		0 (0.0)	30 (11.9)				
Others	0 (0.0)	0 (0.0)		0 (0.0)	9 (3.6)				
**Treatment times**									
< =2	16 (100.0)	36 (97.3)	0.998	2 (28.6)	151 (40.8)	0.980	10 (62.5)	164 (66.1)	0.791
>2	0 (0.0)	1 (2.7)		5 (71.4)	219 (59.2)		6 (37.5)	84 (33.9)	
**Individual income (K RMB)**	55.0 (37.1)	36.0 (20.8)	0.098	50.0 (44.4)	40.0 (29.7)	0.166	43.0 (17.8)	40.0 (24.5)	0.410
**Family income (K RMB)**	100.0 (59.3)	60.0 (44.5)	0.063	150.0 (74.1)	96.0 (80.1)	0.016	100.0 (37.1)	80.0 (57.8)	0.129
**Treatment cost (K RMB)**	10.0 (10.4)	10.0 (12.5)	0.866	2.5 (2.6)	0.7 (0.7)	0.003	—	—	
**Lost income (K RMB)**	1.6 (23.7)	2.5 (2.7)	0.530	0 (0)	0 (0)	0.505	0 (0)	0 (0)	0.276
**Gross total cost (K RMB)**	12.5 (12.4)	16.0 (19.0)	0.993	3.0 (3.4)	1.0 (1.0)	0.014	0.5 (0.3)	0.3 (0.3)	0.219
**Paid by insurance (K RMB)**	5.3 (7.0)	—		0.7 (0.8)	—		0 (0)	—	
**OOP cost (K RMB)**	6.5 (8.5)	16.0 (19.0)	0.063	0.9 (1.3)	1.0 (1.0)	0.933	0.5 (0.3)	0.3 (0.3)	0.585

*For a categorical variable, count (percentage). For a continuous variable with a skewed distribution, median (MAD)*.

Multivariate logistic regression analysis results are presented in Table 3. It is noted that only variables that are significant in the step-wise approach are present in the final models. For inpatient treatment, no variable reaches the 0.05 significance level, and three variables have *p* < 0.1, including education, occupation, and type of household. Compared to the reference group of no schooling, those with junior high education are less likely to use insurance (*OR* < 0.01). Those with hotel and catering occupations are more likely to use insurance (*OR* = 275.06), compared to those in manufacturing. And those with rural hukou are less likely to use insurance (*OR* = 0.32). For outpatient treatment, three variables are identified as significant with the step-wise approach. Specifically, females are more likely to use insurance (*OR* = 13.82), and those in the 50–60 age group (*OR* = 0.13) and those having college and more education (*OR* = 0.03) are less likely to use insurance. For self-treatment, only education has a significant association, with those having college and more education less likely to use insurance (odds ratio 0.13, *p*-value 0.001). It should be recognized that, although all three logistic regression models pass model diagnostics, they may still suffer from small sample sizes and/or highly imbalanced data. As such, although the model fitting may be statistically valid, the results should be interpreted cautiously. For example, in the inpatient treatment analysis, one estimated odds ratio is extremely large, while another is extremely small. Such results may raise alarm.

**Table 3:** Multivariate logistic regression analysis of insurance utilization: estimated odds ratio and *p*-value.

	**Inpatient treatment** ***n =*** **54**		**Outpatient treatment** ***n =*** **269**		**Self-treatment** ***n =*** **379**	
	***OR***	***p***	***OR***	***p***	***OR***	***p***
**Gender (reference: male)**						
Female			13.82	0.022		
**Age group (reference: 18–30)**						
50–60			0.13	0.069		
**Education (reference: no schooling)**						
Junior high	<0.01	0.056				
College and more			0.03	<0.000	0.13	0.001
**Occupation (reference: manufacturing)**						
Hotel and catering	275.06	0.086				
Service						
**Type of household (reference: urban)**						
Rural	0.32	0.066				

### Medical Expenditure

The univariate analysis of total medical and OOP cost is conducted, comparing across groups with different variable values, and the results are presented in Table 4. In the analysis of inpatient treatment, both gross total and OOP costs are found to depend significantly on physical condition. Specifically, the group “seriously sick” has the highest cost, followed by “slightly sick.” Significant differences are also observed for type of hospital. Specifically, using grade III hospitals is associated with the highest cost. For example, the total cost values are 7.0 K (grade II), 24.0 K (grade III), and 2.3 K (private), respectively. More significant variables are observed in the analysis of outpatient treatment. Age is found to be significant, with the >60 group having significantly higher cost. For example, for total cost, those >60 years old have average cost 4.2 K, compared to 2.8 K for the 50–60 group and even lower for the other groups. Physical condition and type of hospital are also found as significant, and the observed patterns are similar to those for inpatient treatment. Another variable found as significant only for total cost is insurance utilization. Specifically, those who used insurance had significantly higher total cost (3.0 vs. 1.0 K). Treatment times is significantly associated with both total cost and OOP cost: those with more treatments are observed to have higher cost. In the analysis of self-treatment, significant variables are age group, physical condition, and treatment times. The observed patterns are similar to those for outpatient treatment.

**Table 4:** Summary statistics of total and OOP cost for different types of treatment (in K RMB).

	**Inpatient treatment**		**Outpatient treatment**		**Self-treatment**	
	**Total cost**	**OOP cost**	**Total cost**	**OOP cost**	**Total cost**	**OOP cost**
**Gender**	*p =* 0.091	*p =* 0.162	*p =* 0.138	*p =* 0.170	*p =* 0.490	*p =* 0.545
Male	21.8 (27.6)	17.3 (24.4)	1.0 (1.0)	1.0 (1.0)	0.3 (0.3)	0.3 (0.3)
Female	10.0 (10.1)	10.0 (12.5)	1.0 (1.2)	1.0 (1.2)	0.3 (0.3)	0.3 (0.3)
**Age group**	*p =* 0.554	*p =* 0.545	*p =* 0.021	*p =* 0.015	*p =* 0.020	*p =* 0.017
18–30	16.0 (20.7)	15.0 (22.1)	0.9 (0.9)	0.8 (0.9)	0.2 (0.2)	0.2 (0.2)
30–40	12.0 (13.9)	7.9 (16.5)	1.2 (1.2)	1.2 (1.2)	0.2 (0.2)	0.2 (0.2)
40–50	18.0 (17.8)	18.0 (25.9)	1.0 (1.0)	1.0 (1.0)	0.4 (0.3)	0.4 (0.3)
50–60	36.0 (51.0)	30.5 (42.9)	2.8 (3.6)	1.6 (1.9)	0.5 (0.6)	0.5 (0.6)
>60	7.0 (4.4)	7.0 (4.4)	4.2 (2.5)	4.2 (2.5)	0.5 (0.3)	0.5 (0.3)
**Marital status**	*p =* 0.379	*p =* 0.338	*p =* 0.849	*p =* 0.690	*p =* 0.238	*p =* 0.262
Single, divorced, widowed	8.2 (11.6)	8.0 (20.8)	1.0 (1.0)	1.0 (1.0)	0.2 (0.2)	0.2 (0.2)
Married	15.5 (16.3)	14.0 (17.8)	1.0 (1.0)	1.0 (1.0)	0.3 (0.3)	0.3 (0.3)
**Education**	*p =* 0.945	*p =* 0.932	*p =* 0.246	*p =* 0.297	*p =* 0.643	*p =* 0.685
No schooling	74.6 (10.6)	24.6 (18.0)	0.9 (0.9)	0.9 (0.9)	0.3 (0.2)	0.3 (0.2)
Primary school	16.0 (17.8)	7.4 (10.2)	1.7 (1.9)	1.7 (1.8)	0.3 (0.4)	0.3 (0.4)
Junior high	21.5 (28.5)	21.5 (28.5)	1.0 (1.0)	0.9 (0.8)	0.3 (0.3)	0.3 (0.3)
Senior high	8.3 (7.6)	8.3 (9.3)	1.0 (1.1)	1.0 (1.0)	0.3 (0.3)	0.3 (0.3)
Junior college	15.0 (20.5)	15.0 (20.5)	1.0 (1.0)	1.0 (1.0)	0.3 (0.3)	0.3 (0.3)
College and more	15.0 (17.9)	7.0 (13.6)	1.0 (1.4)	1.0 (1.3)	0.2 (0.2)	0.2 (0.2)
**Occupation**	*p =* 0.181	*p =* 0.263	*p =* 0.405	*p =* 0.542	*p =* 0.298	*p =* 0.336
Manufacturing	240.0 (348.4)	240.0 (351.1)	0.7 (0.6)	0.5 (0.3)	0.3 (0.3)	0.3 (0.3)
Hotel and catering	12.0 (9.6)	10.2 (13.1)	1.0 (1.0)	1.0 (1.0)	0.3 (0.3)	0.3 (0.3)
Service	13.0 (16.6)	7.6 (10.8)	1.0 (1.1)	0.8 (0.9)	0.2 (0.2)	0.2 (0.2)
Sales	10.0 (8.2)	9.4 (8.2)	1.0 (1.0)	1.0 (1.0)	0.3 (0.3)	0.3 (0.3)
Construction	14.0 (17.8)	1.0 (129.0)	0.4 (0.4)	0.4 (0.4)	0.3 (0.3)	0.3 (0.3)
Unemployed	146.0 (0)	146.0 (0)	1.7 (1.7)	1.7 (1.7)	0.2 (0.2)	0.2 (0.2)
other	275.0 (185.3)	275.0 (185.3)	2.5 (0.7)	1.8 (0.3)	0.7 (0.5)	0.7 (0.5)
**Type of household**	*p =* 0.553	*p =* 0.767	*p =* 0.308	*p =* 0.318	*p =* 0.430	*p =* 0.395
Urban	16.0 (16.3)	10.0 (13.7)	1.0 (1.4)	1.0 (1.3)	0.3 (0.3)	0.3 (0.3)
Rural	13.0 (15.2)	13.0 (25.2)	1.0 (1.0)	1.0 (1.0)	0.3 (0.3)	0.3 (0.3)
**Physical condition**	*p =* 0.001	*p =* 0.002	*p <* 0.001	*p <* 0.001	*p <* 0.001	*p <* 0.001
Healthy	10.0 (7.4)	7.4 (9.5)	0.7 (0.7)	0.7 (0.7)	0.2 (0.2)	0.2 (0.2)
Just so-so	8.5 (10.2)	8.0 (14.8)	2.0 (2.2)	2.0 (2.2)	0.4 (0.3)	0.4 (0.3)
Slightly sick	146.0 (152.7)	146.0 (152.7)	1.5 (1.1)	1.5 (0.8)	0.5 (0.7)	0.5 (0.7)
Sick	40.0 (56.9)	30.0 (42.1)	3.0 (3.5)	3.0 (3.5)	0.8 (0.9)	0.8 (0.9)
Seriously sick	270.0 (44.5)	220.0 (29.7	8.0 (10.9)	8.0 (10.9)	2.0 (2.8)	2.0 (2.8)
**Type of hospital**	*p =* 0.001	*p =* 0.002	*p <* 0.001	*p <* 0.001	—	—
Grade I	—	—	1.0 (1.0)	0.9 (1.0)	—	—
Grade II	7.0 (5.1)	4.0 (6.4)	1.0 (0.9)	1.0 (0.9)	—	—
Grade III	24.0 (30.4)	24.0 (31.7)	2.0 (2.2)	1.6 (1.8)	—	—
Private	2.3 (0)	2.3 (0)	0.4 (0.5)	0.4 (0.4)	—	—
Others	—	—	0.9 (0.4)	0.4 (0.4)	—	—
**Insurance utilization**	*p =* 0.985	*p =* 0.568	*p =* 0.014	*p =* 0.933	*p =* 0.218	*p =* 0.583
No	16.0 (19.0)	16.0 (19.0)	1.0 (1.0)	1.0 (1.0)	0.3 (0.3)	0.3 (0.3)
Yes	12.5 (12.4)	6.5 (84.9)	3.0 (3.3)	0.9 (1.3)	0.5 (0.3)	0.5 (0.3)
**Treatment times**	*p =* 0.280	*p =* 0.281	*p <* 0.001	*p <* 0.001	*p <* 0.001	*p <* 0.001
< =2	14.5 (16.2)	10.0 (14.7)	0.7 (0.6)	0.7 (0.6)	0.1 (0.1)	0.1 (0.1)
>2	170.0 (0)	170.0 (0)	2.2 (2.5)	2.0 (2.4)	0.5 (0.4)	0.5 (0.4)
**Individual income**	*p =* 0.860	*p =* 0.953	*p =* 0.254	*p =* 0.450	*p =* 0.983	*p =* 0.907
< =40 K	12.0 (14.8)	9.0 (10.7)	0.9 (0.9)	0.9 (0.8)	0.3 (0.3)	0.3 (0.3)
>40 K	10.0 (11.9)	9.4 (12.9)	1.0 (1.2)	1.0 (1.2)	0.3 (0.3)	0.3 (0.3)

*For cost (which has a skewed distribution), median (MAD)*.

Multivariate linear regression results are presented in Table 5. With the step-wise approach, only a few variables are found as significant. As the effects of multiple correlated variables are jointly considered, findings different from the univariate analysis are made. In the analysis of inpatient treatment, physical condition and type of hospital are significantly positively associated with cost. In particular, for total cost, with “healthy” as reference, “slightly sick” has estimated regression coefficient 124,000, and “seriously sick” has estimated regression coefficient 218,000. For OOP cost, “seriously sick” has regression coefficient 191,202. For total and OOP cost, using grade III hospital has regression coefficients 18,000 and 94,798, respectively. For outpatient treatment, the 50–60 age group has significantly higher total cost (estimated coefficient 1,271.1). Using grade III hospital is significant for both total and OOP cost (estimated coefficients 600 and 9, respectively). In the analysis of self-treatment, more variables are identified as significant, including age group 40–50 and >60 (for both types of cost), physical condition “just so-so” (for total cost), and number of self-treatment times (for both types of cost). We note that in the analysis of inpatient treatment cost, the estimated coefficients have large magnitudes. A closer examination of data suggests that this is caused by a few subjects with extremely high cost. Although the robust LAD regression technique is adopted, with the overall small sample size, these subjects still seem to have a high impact on estimation. As such, the findings should be interpreted cautiously.

**Table 5:** Multivariate linear regression analysis of total and OOP cost for different types of treatment: estimated coefficient and *p*-value.

	**Inpatient treatment**				**Outpatient treatment**				**Self-treatment**			
	***n =*** **54**				***n =*** **269**				***n =*** **379**			
	**Total cost**		**OOP cost**		**Total cost**		**OOP cost**		**Total cost**		**OOP cost**	
	**Estimate**	***p***	**Estimate**	***p***	**Estimate**	***p***	**Estimate**	***p***	**Estimate**	***p***	**Estimate**	***p***
**Age group (reference: 18–30)**												
40–50									6.2	0.007	7.0	0.0005
50–60					1271.1	0.049						
>60									13.0	0.018	13.0	0.011
**Physical condition (reference: healthy)**												
Just so-so									5.0	0.035		
Slightly sick	124000.0	0.021										
Seriously sick	218000.0	0.018	191202.0	0.050								
**Type of hospital**												
Grade III	18000.0	0.002	94798.0	0.004	600.0	0.034	9.0	0.046				
**Self-treatment times**	—	—	—	—	—	—	—	—	10.8	0.001	11.2	0.003

## Discussions

It has been suggested in the literature that the floating population in China, as well as their counterparts—the migrant workers in other countries, are disadvantageous in healthcare. Our literature search suggests that, for China's floating population, most of the existing studies have focused on the managerial and philosophical aspects, or a single type of disease/treatment. This study can complement the existing studies and fill the knowledge gap by analyzing empirical data directly collected from the floating populating and describing multiple aspects of healthcare including demographic characteristics of those under care, insurance utilization, and medical expenditure, all of which are of critical interest to public health researchers, healthcare providers, and other stakeholders.

The constrained financial and human resources have led to the small sample size, which poses a major limitation to this study. Nevertheless, it should be recognized that in the literature, multiple important findings have been made based on data with limited sample sizes. For example, studies had recruited a total of 475 migrant workers in Shanghai to study the migration stress, prevalence of mental disorders, and socio-demographic correlates of mental health (33, 34), and findings with critical importance for the prevention of mental illness in migrant workers were made. Studies such as Hiott et al. (35), Price et al. (36), and Holmes (37) all have sample sizes smaller than 200, however, had generated important findings on migrant workers in the U.S. Another limitation is that data collection was limited to Beijing. Published studies, including the aforementioned, have suggested that valid findings can still be generated when data collection is geographically limited. In addition, literature has suggested that differences between migrant workers in different cities are considerably smaller than those for residents.

In this study, data was only collected on the floating population, without a general population comparison group. A qualitative comparison has been made against the population summary data for the city of Beijing published by the Bureau of Statistics of China (www.stats.gov.cn/tjsj/ndsj/2014/indexch.htm; it is noted that the present study and that by the Bureau of Statistics may have different sampling schemes, and as such, a quantitative comparison may not be sensible). Compared to the general population, the floating population has multiple unique characteristics. Specifically, they are relatively younger, less educated, with a lower income, and have labor-intensive jobs, which are in general associated with lower socioeconomic status. Some of those characteristics have been identified as associated with disadvantageous healthcare in the literature (10, 38–40). For example, occupation has been identified as associated with pursuing healthcare for the floating population. Many occupations that the floating population has are associated with long working hours (including night and weekend shifts) and no paid time off, which create barriers for hospital-based healthcare (18). A published cross-sectional study showed that workers with a lower level of education were more likely to pay higher insurance agent fees and have poorer understanding of what was covered by insurance. In addition, it was also found that employers were more likely to pay insurance contributions for more advantaged workers (with more experience, more stable, male, and better educated) but not for less advantaged including migrant workers (41). Consistent with the literature, the finding on education suggests that improving education level and promoting health knowledge may eliminate the barrier to health care and health insurance for the floating population. In an audit study, it was reported that migrant workers with low salary often found it challenging to raise enough money for hospital cost even if they were insured, and “unable to pay” was identified as a major reason for not pursuing inpatient or outpatient care (32). In another study, the frequency of migrant workers visiting hospitals was associated with age, gender, insurance, and work type (40). As discussed in the literature and also observed in our study, compared to the general population, the floating population is more poorly covered by health insurance at their residence location, which can have a significant adverse impact on their health conditions and financial consequences (of illness conditions). On the other hand, we also note that with multiple unique characteristics, some findings on the floating population can differ significantly from the general population. For example, in a study on the general population conducted in China, it was found that those who had chronic diseases, earned higher income, resided in urban areas, lived in the middle or eastern regions, or lived in households with the household heads having a middle school or higher education paid more for healthcare (42). However, such findings are not made in this study. With respect to the utilization of health insurance, Liu and Zhao (43) found that it had significantly increased the utilization of formal medical services but had not reduced OOP health expense. The latter finding is consistent with ours. The analysis of CHARLS (China Health and Retirement Longitudinal Study) data suggested that people with lower income and lower level of education, older and divorced/widowed women, as well as rural-registered people had a lower probability of being insured (44). Some of these factors (gender, education, and income) have also been identified in our analysis.

For inpatient treatment—the type usually corresponding to the worst illness condition and highest cost, the leading condition is trauma, which is often work-related. The floating population usually work in labor-intensive jobs, and the high frequency of work-related illness conditions has been observed in the literature. For the studied cohort, the 18–30 age group dominates, leading to childbirth as the second highest inpatient treatment condition. With the unique demographic and occupational characteristics of the floating population, the most prevalent illness conditions and distribution differ from the general population (which, for example, may have a higher rate of aging related illness). For outpatient and self-treatments, similar plausible explanations hold.

The observed insurance coverage condition fits the unique characteristics of the floating population. Specifically, as the majority of the floating population are from rural, at their hometown, they are mostly covered by the NCMS. At Beijing, as most of the floating population are employed, the dominating insurance category is the UEBMI. In contrast, for the general Beijing and other urban population, the dominating categories are UEBMI and URBMI. It is observed that at Beijing, the insurance coverage rate is significantly lower than that of Beijing residents. With the poor portability (of insurance at their hometown), the floating population is not well-protected and vulnerable. Certain misconceptions on insurance, such as “insurance is useless,” are observed. Overall, our findings suggest that the current insurance system needs further improvement. Specifically, portability needs to be improved to facilitate insurance utilization by the floating population (and others) at their live/work places. This needs to be achieved by modifying/removing the pre-approval procedure and allowing for requesting reimbursement at the locations of treatment. In addition, better, and more targeted educational programs are needed. Considering the usually low education level of the floating population, easy-to-comprehend educational materials and delivery mechanism are needed. The insurance system also needs to improve in terms of increasing coverage depth and simplifying procedures.

It is noted again that in the analysis of insurance utilization, with the small sample sizes and highly imbalanced data, the multivariate regression analysis results may need to be interpreted with cautions. As such, conclusions have been mostly drawn from the univariate analysis, which can be more reliable. It is observed that the insurance utilization rate is significantly lower than that of the general population. Comparing the coverage and utilization rates suggests that a considerable percentage of the floating population were covered but did not use insurance. This unique phenomenon of “had but did not use insurance” has been studied in the literature. Results in Figure 1E suggest that the current depth of insurance coverage needs further improvement (to address the “disease not covered” problem), and the insurance utilization procedure needs further simplification (to address the “insurance too complicated to use” problem). The behavior of health insurance utilization, although has been noted in the literature, has not been well-studied, especially for the floating population. Multiple factors have been identified as significantly different between groups with different insurance utilization status. For inpatient and outpatient treatments, education is found as significant, which has also been suggested in published studies (39, 45). Education level has been suggested as playing an important role in healthcare pursuit behaviors in general (46, 47). Different types of treatment differ significantly, in terms of corresponding sample characteristics, illness conditions, and others. For outpatient treatment, occupation and financial conditions have also been observed as significant. Both factors reflect socioeconomic status, whose significance in healthcare pursuit has been well-documented. In the analysis of self-treatment, the especially small sample size may contribute to the lack of significance. Further data collection is needed. Insurance is not effective unless used. Our findings can assist insurance agencies, healthcare providers, and other stakeholders identifying subgroups with especially low insurance utilization. Interventions need to be developed targeting those groups to improve utilization.

In the literature, research on medical expenditure of the floating population is much less compared to that for the general population. This study can partly fill this knowledge gap. Compared to the existing studies based on hospital data, this study can be advantageous by also having information on self-treatment. Although the cost of self-treatment per episode is low, with the high frequency, the accumulated cost should not be ignored. This study is among the first to provide comprehensive and separate information on all three types of treatment for the floating population. It is observed that the medical expenditure level for those with inpatient treatment is especially high (average 14.75 K, compared to 40.00 K of individual income—a qualitative literature review suggests that this ratio can be higher than that for the general population). Combined with the low insurance utilization rate, the high medical cost can lead to severely adverse financial and other consequences (48). Beyond further improving insurance portability and utilization, insurance, and healthcare providers also need to further improve coverage depth and reimbursement rate to reduce financial burden to patients. The cost of outpatient and self-treatments is much lower, however, can still pose serious concerns given the low income level. It has been recognized in multiple published studies that self-treatment is poorly covered by insurance. However, as self-treatment is not hospital-based and hard to administratively manage, it is still unclear how to reduce self-treatment-related cost. Multiple factors have been found as associated with the levels of cost for the three types of treatment. Both age group and physical condition have intuitive interpretations and have also been observed in the literature (46). Type of hospital has been found as significant in univariate analysis but not multivariate analysis. Grade III hospitals offer the highest level of care and often treat the most serious illness conditions, both contributing to the high cost. In multivariate analysis, the small number of significant variables can be attributable to confounding (for example, with physical condition) in addition to the small sample size. Among the identified significant variables, age, and physical condition are directly related to health conditions. It is noted that they may also be confounded with insurance utilization and other factors. The higher cost associated with using Grade III hospitals has also been observed in the literature and has a simple interpretation. Similar holds for the number of self-treatment times. For the general population and other sub-populations, all these variables have been suggested in some studies as relevant, although there is still a lack of full consensus. Some published studies have larger sample sizes and have identified other/more variables as associated with cost. Our findings may assist researchers and healthcare providers better understanding the healthcare characteristics and medical expenditure structure of the floating population, which are lacking in the literature. The distribution of medical expenditure is not uniform across people. Identifying those with higher cost may assist the implementation of targeted effort to reduce cost.

## Limitations

The most prominent limitation is that, with limited financial and human resources, data collection has been limited. This is manifested in multiple aspects. In particular, the sample size is small. However, as previously discussed, many of the existing studies have been able to generate important findings based on comparable or even smaller sample sizes. In addition, the collected samples have a wide range of demographic and personal characteristics, providing a wide spectrum of information. Secondly, certain information, such as cultural and religious information, has not been collected. However, we have arguably collected the most crucial information as suggested by the published literature. Thirdly, there is a lack of data on the general population, which prevents a direct and quantitative comparison. Nevertheless, we have been able to make qualitative comparisons with the general population and findings in the literature. All information has been collected through survey, and the quality of survey data has been discussed in multiple publications. The study has a cross-sectional nature, which inevitably has limitations. For example, causal relationships cannot be inferred, and possible change over time cannot be analyzed. On the other hand, it is noted that cross-sectional survey is still very commonly used in the study of healthcare, insurance, and expenditure. The aforementioned limitations are also shared by many published studies, which have convincingly established merits of such survey data/research.

## Conclusions

For the large-sized but little-investigated floating population, we have conducted a survey with a focus on their healthcare. Demographic characteristics of those under care have been provided, which may assist better describing and understanding this unique population. It is found that the floating population has low insurance coverage and utilization but high medical cost. Policy interventions are needed to improve the portability of health insurance, and targeted and better education is needed to improve the understanding of health insurance utilization, so as to improve insurance coverage/utilization and reduce financial burden. Demographic characteristics including gender, age, education, occupation, and type of household have been identified as associated with insurance utilization. Age, physical condition, type of hospital, and self-treatment times have been identified as associated with medical cost. The identified significant factors can assist identifying the especially disadvantaged in the floating population, and the estimated odds ratios and regression coefficients can help prioritize these factors. Such results can help policy implementation be more targeted. Overall, with the importance of the floating population and lack of research, our findings can be valuable to healthcare providers, health insurance policymakers, public health researchers, and others. It is finally noted that the healthcare and insurance systems in China are evolving fast. Findings made in this study may need update in the near future.

## Data Availability Statement

The datasets presented in this study can be found in online repositories. The names of the repository/repositories and accession number(s) can be found below: https://figshare.com/s/4a1ee991a045e35e8030.

## Ethics Statement

The study was approved by an ethics review committee at the Renmin University of China. The patients/participants provided their written informed consent to participate in this study.

## Author Contributions

XW and SM designed the study. YL, YZ, YJ, and YW designed the survey. CM conducted data analysis. CM and SM drafted the manuscript. All authors read and approved the final version of the manuscript.

## Conflict of Interest

The authors declare that the research was conducted in the absence of any commercial or financial relationships that could be construed as a potential conflict of interest.

## Abbreviations

UEBMI: Urban Employee Basic Medical Insurance, for the employed in the urban areas
URBMI: Urban Resident Basic Medical Insurance, for urban residents not covered by the UEBMI
NCMS: New Rural Cooperative Medical Scheme, for the rural residents
CSPH: China Survey on Pension and Healthcare.
